# Basic science knowledge underlies clinical science knowledge and clinical problem solving: evidence from veterinary medicine

**DOI:** 10.1007/s10459-024-10334-2

**Published:** 2024-05-16

**Authors:** Jared A. Danielson, Rebecca G. Burzette, Misty R. Bailey, Linda M. Berent, Heather Case, Anita Casey-Reed, John Dascanio, Richard A. Feinberg, Tamara S. Hancock, Claudia A. Kirk

**Affiliations:** 1 Department of Veterinary Pathology, Iowa State University, Ames, IA USA; 2 College of Veterinary Medicine, Iowa State University, Ames, IA USA; 3 College of Veterinary Medicine, University of Tennessee, Knoxville, TN USA; 4 Department of Veterinary Pathobiology, University of Missouri, Columbia, MO USA; 5 International Council for Veterinary Assessment, Bismarck, ND USA; 6 College of Veterinary Medicine, Lincoln Memorial University, Harrogate, TN USA; 7 National Board of Medical Examiners, Philadelphia, PA USA; 8 School of Veterinary Medicine, Texas Tech University, Amarillo, TX USA

**Keywords:** Assessment, Clinical learning, Encapsulated knowledge, Standardized exams

## Abstract

Medical sciences education emphasizes basic science learning as a prerequisite to clinical learning. Studies exploring relationships between achievement in the basic sciences and subsequent achievement in the clinical sciences generally suggest a significant positive relationship. Basic science knowledge and clinical experience are theorized to combine to form encapsulated knowledge– a dynamic mix of information that is useful for solving clinical problems. This study explores the relationship between basic science knowledge (BSK), clinical science knowledge (CSK), and clinical problem-solving ability, as measured within the context of four veterinary colleges using both college-specific measures and professionally validated, standardized measures of basic and clinical science knowledge and problem-solving ability. Significant correlations existed among all variables. Structural equation modeling and confirmatory factor analysis were used to produce models showing that newly acquired BSK directly and significantly predicted BSK retained over time and newly acquired CSK, as well as indirectly predicted clinical problem-solving ability (mediated by newly acquired CSK and BSK retained over time). These findings likely suggest a gradual development of schema (encapsulated knowledge) and not an isolated development of biomedical versus clinical knowledge over time. A broader implication of these results is that explicitly teaching basic science knowledge positively and durably affects subsequent clinical knowledge and problem-solving ability independent of instructional strategy or curricular approach. Furthermore, for veterinary colleges specifically, student performance as measured by both course-level and standardized tests are likely to prove useful for predicting subsequent academic achievement in classroom and clinical settings, licensing examination performance, and/or for identifying students likely in need of remediation in clinical knowledge.

## Introduction

Research across multiple disciplines supports the notion that conceptual, verbal, and principle-based, discipline-specific knowledge is foundational for problem solving in any knowledge domain (Jonassen, 2000). This assumption is inherent in many current curricular approaches to medical sciences education, which emphasize basic science learning as a prerequisite to clinical learning (e.g. Finnerty et al., 2010). Despite the general assumption that basic science knowledge undergirds clinical learning and/or proficiency, research in the medical sciences has produced a variety of perspectives regarding the relationship between basic science knowledge and subsequent clinical proficiency, which is manifested in tasks such as making an accurate diagnosis and treatment plan. Furthermore, answering the question of how most effectively to integrate basic science knowledge into curricular programs meant to produce clinical proficiency remains elusive (Kulasegaram et al., 2013). This challenge is not unique to training human physicians, and is increasingly receiving attention in veterinary medical education as well. Particularly as Competency Based Veterinary Medical Education (AAVMC et al., 2018a, 2018b; Salisbury et al., 2019) has received increased attention, those who teach topics in the basic sciences have struggled to define their role in competency based educational models.

Researchers have employed three broad strategies to explore how basic science knowledge relates to clinical science proficiency: (1) Observing experts to determine their clinical reasoning processes, or comparing clinical reasoning between novices and experts (Rikers et al., 2005; Rikers, Schmidt et al., 2005); (2) Teaching novices in a variety of ways, some of which rely on basic science knowledge, and some of which do not (Baghdady et al., 2009, 2013; Woods et al., 2005, 2006, 2006b, 2007); and (3) Analyzing the relationship between basic and clinical science knowledge across time as clinical proficiency grows (Schauber et al., 2013).

Multiple studies show that medical experts employ more biomedical knowledge than less experienced practitioners, even though that knowledge may be encapsulated within their clinical knowledge, and, therefore, difficult to detect (de Bruin et al., 2005; Norman et al., 1989; Rikers et al., 2004; Rikers, Loyens, Rikers et al., 2005a; Rikers, Schmidt et al., 2005). For instance, de Bruin et al. (2005) administered tests of clinical diagnosis ability, basic science knowledge, and clinical knowledge to family physicians and medical students at various points in their training. They found that, for both students and physicians, clinical knowledge mediated the predictive relationship between basic science knowledge and diagnostic performance, meaning that basic science knowledge predicted diagnostic performance when it also predicted clinical knowledge. This mediated relationship can be interpreted to mean that basic science knowledge contributed to clinical problem solving ability particularly for those individuals for whom it had also contributed to clinical knowledge.

Similarly, in studies exploring the relationship between the extent to which instruction employs basic science concepts and subsequent proficiency, educational strategies that integrate basic science and clinical concepts produce greater learning gains than those that do not (Baghdady et al., 2009; Kulasegaram et al., 2013, 2017; Lisk et al., 2016; Woods et al., 2005, 2006, 2006b, 2007). For example, Baghdady and colleagues (2009), found that when pre-dental students were taught basic science concepts, they performed diagnostic tasks more accurately than students who were taught structured algorithms or feature lists, even though all students performed similarly on a simple memory test of the facts that had been taught.

Studies exploring relationships between achievement in the basic sciences and subsequent achievement in the clinical sciences generally, but not universally, suggest a significant positive relationship. Cianciolo and colleagues (2013) found a low-moderate positive relationship between biomedical knowledge and clinical information gathering and interpretation in medical students over time. Similarly, studies have shown that basic science knowledge during veterinary school (Danielson et al., 2011) and prior to veterinary admission (Danielson & Burzette, 2020) significantly and positively predicted clinical problem-solving ability as measured by licensing examination scores. In contrast, Schauber et al. (2013) found a negative relationship between students’ early levels of basic science knowledge and subsequent gains in clinical knowledge. They speculated that this unexpected finding could be due to individual differences in student motivation or cognitive characteristics, interference from irrelevant information, or inappropriate transfer.

One complexity associated with discussions of basic science and clinical science knowledge are the variety of labels that have been employed to refer to the many intellectual skills that contribute to clinical proficiency. Labels such as “knowledge,” “reasoning,” “critical thinking,” and “problem solving” can all be found in the literature, but researchers do not use these labels equivalently. Furthermore, there is a paucity of studies establishing psychometrically measurable distinctions between closely associated intellectual skills such as “diagnostic reasoning,” “clinical reasoning,” “critical thinking,”, “diagnostic problem solving” and “clinical problem solving.” The present study does not seek to resolve this dilemma or to provide a typology of intellectual skills in the medical sciences. However, in order to provide definitional clarity to constructs measured in the present study, we define those constructs as follows, borrowing from Smith and Ragan’s (2005) taxonomy of learning outcomes, which were adapted from Gagné et al’s (1992) varieties of learning:

### Basic science knowledge

We define basic science knowledge as all of the declarative knowledge, concepts, principles and procedures associated with the disciplines of anatomy, physiology, pathology, microbiology, and pharmacology that underly a veterinary medical education. As specified by the AVMA COE accreditation standards, this knowledge provides “an understanding of the central biological principles and mechanisms that underlie animal health and disease from the molecular and cellular level to organismal and population manifestations” and “scientific, discipline-based instruction in an orderly and concise manner so that students gain an understanding of normal function, homeostasis, pathophysiology, mechanisms of health/disease, and the natural history and manifestations of important animal diseases...” (AVMA-COE, 2023 p. 25).

### Clinical science knowledge

We define clinical science knowledge as all of the declarative knowledge, concepts, principles, procedures, and problem-solving ability associated with the theory and practice of medicine. As defined by the AVMA COE accreditation standards, this knowledge includes “principles and hands-on experiences in physical and laboratory diagnostic methods and interpretation (including diagnostic imaging, diagnostic pathology, and necropsy), disease prevention, biosecurity, therapeutic intervention (including surgery and dentistry), and patient management and care (including intensive care, emergency medicine and isolation procedures) involving clinical diseases of individual animals and populations,” and emphasizing “problem solving that results in making and applying medical judgments” (AVMA-COE, 2023 pp. 25–26).

### Clinical problem-solving ability

Smith and Ragan define problem solving as the ability to “select from a number of possible rules, whether relational or procedural, and apply those rules in a unique sequence and combination to solve a previously unencountered problem” (2005 p. 81). For the purpose of the present study, scores on the North American Veterinary Licensing Examination (NAVLE®) were used as a measure of clinical problem solving. The NAVLE® provides realistic clinical scenarios that require examinees to synthesize their existing knowledge and select an appropriate diagnosis or next step related to diagnosis or treatment. Thus, the content of NAVLE is focused on clinical knowledge, but the item format often requires problem solving skills that go beyond factual recall to correctly respond.

Theoretically, basic science knowledge contributes to clinical knowledge and/or clinical problem-solving ability in two ways. First, as noted above, multiple studies have established that when students learn clinical concepts in the context of underlying basic science knowledge, they are better able to solve clinical problems (Baghdady et al., 2009, 2013; Kulasegaram et al., 2013, 2017; Lisk et al., 2016; Woods et al., 2005, [Bibr CR37][Bibr CR38], 2007). In the studies cited, this effect was evident within a week of initial exposure to the content.

Second, underlying biomedical knowledge has been shown to be inherent to clinical problem solving in experts. Castillo et al. summarize the contribution of basic science knowledge to clinical ability in experts as follows:

“For the experienced physician, biomedical knowledge is sometimes described as encapsulated with clinical knowledge in mental representations of diseases (Schmidt & Rikers, 2007). This clustering of symptoms into meaningful patterns based on basic science knowledge (Schmidt & Boshuizen, 1992) provides a way of explaining symptoms simultaneously, thus facilitating clinical problem solving (de Bruin et al., 2005; Schmidt & Boshuizen, 1993). Therefore, the value of the basic sciences in clinical reasoning goes beyond the development of static knowledge structures. Rather, basic science knowledge should also serve as the foundation for the development of dynamic mental structures to support medical problem solving” (Castillo et al., 2018 p. 593).

From this perspective, the expert’s dynamic, encapsulated knowledge comprises both biomedical knowledge and clinical knowledge (characterized by exemplars encountered in practice)– and is accessed dynamically when clinical problems are encountered (Rikers, Schmidt, et al., 2005).

If basic science knowledge contributes to clinical knowledge in the ways described above, measures of basic science knowledge should predict measures of clinical ability for at least two reasons. First, basic science knowledge, independent of clinical experience or encapsulated knowledge, provides a foundational set of concepts and principles upon which learners draw as they solve clinical problems; therefore, measures of basic science knowledge should directly predict clinical science knowledge and/or ability. Second, basic science knowledge and clinical experience are theorized to combine to form encapsulated knowledge– a dynamic mix of information that is useful for solving clinical problems. Therefore, as learners grow in knowledge and experience, their maturing knowledge structures begin to resemble, even if only modestly at first, the encapsulated knowledge of experts. Such knowledge structures would begin to form as students first encounter clinical cases and should be reflected by grades in clinical courses. That being the case, the relationship between foundational basic science and clinical problem-solving ability should also be mediated by intermediate measures of clinical science knowledge and ability. Finally, if basic science knowledge persists over time, independently of encapsulated knowledge, a strong correlation should exist between basic science knowledge when it is first acquired and basic science knowledge that is retained over a period of months to years. Furthermore, because memories fade over time (Anderson, 2000), basic science knowledge that has been retained over time, either because it has been rehearsed, or because it has been encapsulated in clinical science knowledge, should predict subsequent clinical proficiency more powerfully than basic science knowledge when it is first acquired.

It is important to note that these relationships between basic science knowledge and subsequent clinical science knowledge and proficiency do not infer a particular curricular strategy or sequence. For example, some curricula introduce basic science principles early on with little discussion of clinical application. Other curricula integrate basic science instruction into clinical instruction very early in the educational process. Hypothesized relationships between basic science knowledge and clinical science knowledge and proficiency would, theoretically, be consistent across a variety of curricular approaches.

The present study explores the relationship between basic and clinical science knowledge as measured within the context of four veterinary colleges using both college-specific measures and professionally-validated, standardized measures of basic science knowledge, and clinical problem solving.

**Fig. 1:**
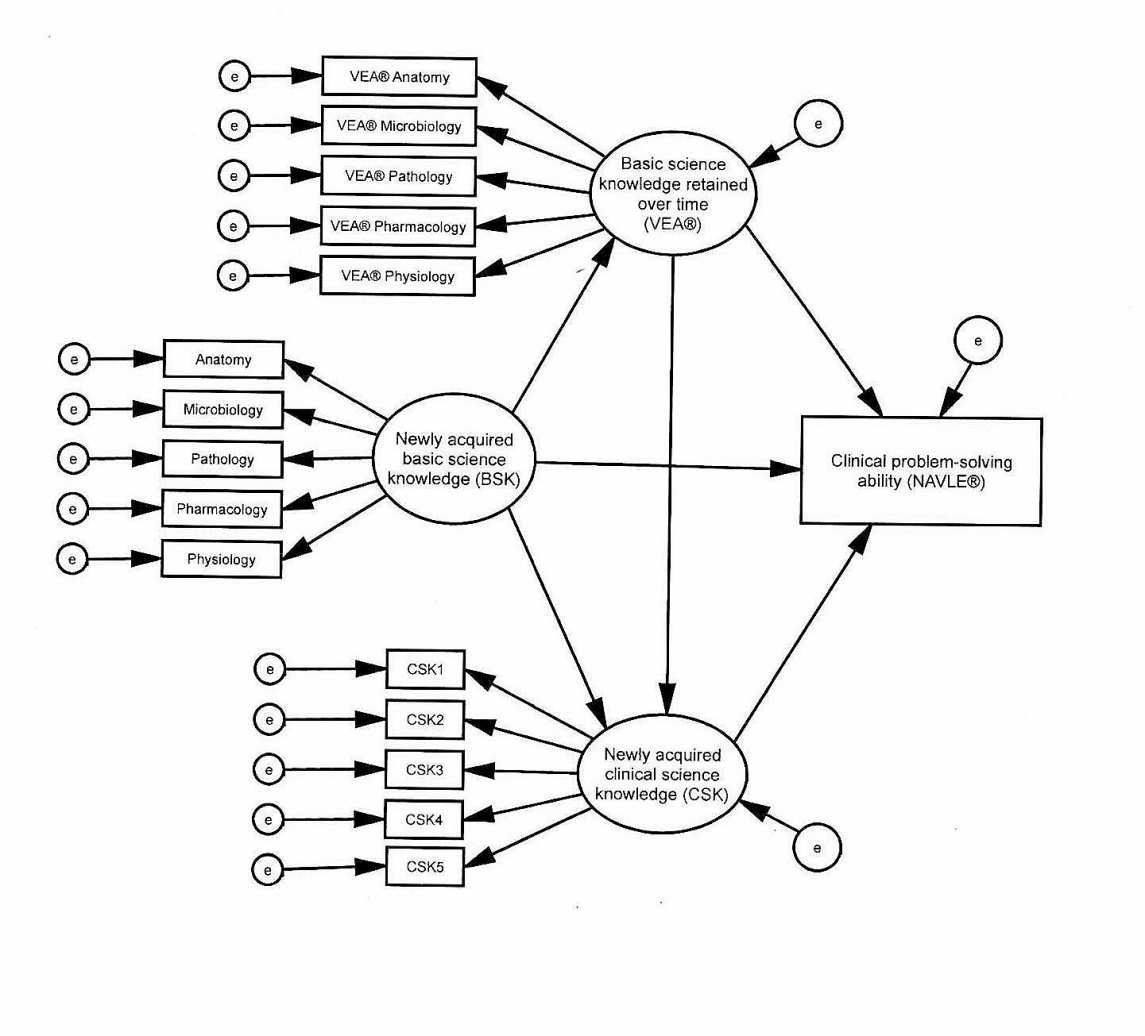
Hypothesized model. Newly acquired basic science knowledge (BSK) represented course grades in the subjects shown. Newly acquired clinical science knowledge (CSK) was measured with student achievement in clinical courses. e = error, VEA® = Veterinary Educational Assessment, NAVLE® = North American Veterinary Licensing Examination

The model shown in Fig. 1 tests the hypothesized relationships discussed above as measured in the present study. The measures, described in more detail in the Materials and Methods section, were as follows: (1) Measures of basic science knowledge included grades in basic science courses and the Veterinary Educational Assessment (VEA®); (2) Grades in clinical science courses were used to measure clinical science knowledge; and (3) Scores on the North American Veterinary Licensing Examination (NAVLE®) were used as measures of clinical problem-solving ability. We hypothesized that pooling basic sciences grades would produce a valid basic science knowledge construct, pooling clinical science grades would produce a valid clinical science knowledge construct, and that the sub scores of the VEA would also form a valid construct representing basic science knowledge retained over time. We proposed testing these hypotheses with confirmatory factor analysis.

Given the documented relationship between basic science knowledge and clinical ability, regardless of expertise level, we hypothesized a positive predictive relationship between measures of basic science knowledge and subsequent measures of clinical knowledge and problem-solving ability (direct effects). Given the assumption that encapsulated basic science knowledge contributes to problem-solving ability, we hypothesized that the relationship between basic science knowledge and clinical problem-solving ability would be mediated by clinical science knowledge (specific indirect effects). Finally, we theorized a significant positive relationship between basic science knowledge shortly after it was acquired and basic science knowledge that was retained over time (direct effect). We proposed using structural equation modeling to test these hypotheses.

## Materials and methods

### Participants

Students from four AVMA-COE accredited colleges of veterinary medicine, two located in the midwest and two in the southeast of the United States were study participants. All participating students graduated between 2016 and 2019. During the period of the study, one of the participating institutions administered the VEA® four times, two administered it three times, and one administered it twice, with 1,310 students total taking both the VEA® and NAVLE® exams. Of the students taking both exams, 1,161 (88.6%) consented to have their NAVLE® scores released to their college, and were included in the study. Participants were 79.9% female and 20.1% male, in response to a male/female binary questionnaire item; ages at the time of the VEA® administration ranged from 22 to 53 years (*M* = 25.8, *SD* = 2.71).

### Procedure

This study was approved by the Iowa State University Institutional Review Board (exempt; IRB #18-438-01). The data were collected from academic records for students who graduated between 2016 and 2019 from one of four U.S.-based veterinary programs accredited by the American Veterinary Medical Association Council on Education (AVMA-COE). All students participated in the curriculum of one of the four participating colleges. Two of the colleges employed a traditional, discipline-based curriculum in which students participated in predominantly lecture and laboratory instruction for 3 years, with early semesters focusing on the basic sciences and later semesters focusing more on the clinical sciences (such as medicine and surgery). During the fourth year, students learned in clinical rotations where they spent most of their time working directly with client-owned animals under the direct supervision of veterinary faculty within a veterinary teaching hospital. The curriculum of the third institution was similar, except that didactic instruction was completed in 2 years (including summers) and followed by 2 years of clinical instruction. The curriculum at the fourth institution was similar to the first two, except that the fourth-year clinical instruction was offered in preceptorships in a variety of privately-owned clinics, rather than in an institutionally-owned teaching hospital.

### Measures

Evaluation practices differ among instructors, with some basing grades exclusively on individuals’ exam scores, and others including other factors such as quiz scores, group participation, assignments, or extra credit experiences. Therefore, in order to minimize the potential effect of factors that influenced student grades, but did not reflect achievement, for basic or clinical science courses, instructors were asked to select the best indicator of overall student achievement, with some choosing the course grade, and others choosing one or multiple pooled exam scores. For each course, the instructor’s chosen student achievement measure functioned as the *grade* for the purposes of this study. Course grades were designated as either basic science (courses in anatomy, physiology, pathology, microbiology, and pharmacology) or clinical science (courses emphasizing the practice of medicine or surgery). For all participating students, grades in basic and clinical sciences courses were collected, as well as VEA® and NAVLE® scores. A more detailed description of each measure or category of measures is provided below.

*Newly acquired basic science knowledge (BSK)* Basic science grades were computed for each content area that mirrored those assessed in the VEA® (anatomy, microbiology, pathology, pharmacology, and physiology). For instance, if a student took two courses in anatomy, one course in pharmacology, three microbiology-related courses, and two courses in pathology, the average grade for each cluster of courses would represent that student’s mean score for that content area. We refer to these scores as newly acquired basic science knowledge (BSK) because they measure what students recalled directly after studying for a course in which they were enrolled. Certainly, much of the knowledge that contributed to such grades had been acquired weeks or even months prior to the assessments that produced them. However, tested/graded knowledge is almost invariably reviewed by examinees shortly before an assessment.

For each college, we computed mean grades for each basic science course area, based on the areas assessed in the VEA® (anatomy, microbiology, pathology, pharmacology, and physiology). These variables served as indicators for newly acquired BSK. Note that these indicators were not identical across colleges.

*Basic science knowledge retained over time (VEA®)* The VEA®, developed by the International Council for Veterinary Assessment (ICVA), is a 240-item, multiple-choice examination that was designed to assess knowledge in the areas of veterinary anatomy, physiology, pharmacology, microbiology, and pathology (ICVA, 2022). At the time it was administered to the participants in this study, the VEA® included 200 items. As is standard for commercially available standardized examinations, the VEA® was administered in multiple forms, with different forms used each time the examination was administered.

For the present study, VEA® scores are considered to represent knowledge retained over time rather than newly acquired knowledge because the VEA® measured knowledge that had persisted well beyond the point at which it was assessed for a grade. All participants took the VEA® in the third year of their veterinary training, months to years (depending on the topic) after completing all, or the overwhelming majority, of their basic science curriculum.

*Newly acquired clinical science knowledge (CSK)* Each institution provided grades for all core clinical science (e.g., medicine or surgery) courses (approximately 20 per institution), including both didactic courses and clinical rotations. For each institution, all clinical science courses were randomly assigned to one of five clinical science indicators (CSK1– CSK5), with each indicator comprising scores from four to five courses, and each indicator’s value being the average grade for the randomly-assigned courses. As was the case with basic science course grades, these scores are considered to represent newly acquired knowledge because they were earned directly following study. While the participating colleges all offered similar clinical science courses, such as medicine and surgery, all of their courses were unique to their colleges, so none of the indicators were identical across colleges, however, they were hypothesized to measure equivalent constructs, and therefore, were treated equivalently in the data modeling. Their hypothesized equivalence was tested by determining whether or not they produced consistent results in the model.

*Clinical problem-solving ability (NAVLE®)* The NAVLE® is a 360-item, multiple-choice examination administered by the ICVA that is required for veterinary licensure in the United States and Canada. Like the VEA®, the NAVLE® was professionally developed adhering to rigorous psychometric standards. Students are not required to release their NAVLE® scores to their schools. Approximately 91% of students released their NAVLE® scores to the four colleges in the study timeframe (A. Casey-Reed, personal communication, August 13, 2021).

### Analytic strategy and data modeling

Within each college, the grade-based indicators as well as the VEA® and NAVLE® scores were standardized, then collapsed across colleges.

Correlations were calculated among all variables to show zero-order relationships in order to allow subsequent replication of the analysis, and to aid in interpretation of the subsequent structural equation model.

We used structural equation modeling (SEM) to test relationships among the variables of interest. Structural Equation Modeling was used because it allows for exploration of mediated relationships among variables, and we hypothesized mediated relationships as students’ knowledge increased over time. Confirmatory factor analysis (CFA) was used to verify the measurement quality of the latent constructs used in the model, with standardized and unstandardized coefficients, standard error, probability value, and squared multiple correlation all being calculated and reported. As part of the SEM, Mahalanobis distances were calculated to identify multivariate outliers, which can lead to spurious results. Inspection of Mahalanobis distances in the initial sample of 1,177 identified 16 cases that were multivariate outliers (*p* <.001). These cases were excluded from further analysis, leaving 1,161 cases. We then examined the distributions of the indicators; no variable showed excessive skewness (< 2.1) or kurtosis (< 7.0) (see Table 1).

Goodness of fit indices were examined. Thresholds for a good fit were root mean square error of approximation (RMSEA) < 0.08, comparative fit index (CFI) > 0.95, Tucker-Lewis fit index (TLI) > 0.95, and standardized root mean square residual (SRMR) < 0.08 (Hu & Bentler, 1999). The maximum likelihood method was used to estimate parameters because the data were normally distributed.

All analyses were conducted using IBM SPSS 27 and IBM SPSS Amos 27.

## Results

### Correlations

Table 1 summarizes correlations. We expected correlations among measures of basic science skills and among measures of clinical science skills to be higher than those between measures of basic science skills and clinical science skills. Inspection of Table 1 shows that correlations among the basic science measures ranged from 0.721 to 0.815; correlations among the clinical science measures ranged from 0.508 to 0.616; correlations among the VEA® measures ranged from 0.437 to 0.673. Correlations between the basic science measures and the clinical science measures ranged from 0.521 to 0.678; correlations between the basic science measures and the VEA® measures ranged from 0.330 to 0.501; correlations between the clinical science measures and the VEA® measures ranged from 0.243 to 0.366; correlations among the basic science measures and the NAVLE® ranged from 0.567 to 0.626; correlations among the clinical science measures and the NAVLE® ranged from 0.469 to 0.579; correlations among the VEA® measures and the NAVLE® ranged from 0.463 to 0.580.

**Table 1:** Significant correlations for confirmatory factor analysis and structural equation modeling analyses

	1	2	3	4	5	6	7	8	9	10	11	12	13	14	15	16
1. Anatomy (BSK)	--															
2. Microbiology (BSK)	0.777	--														
3. Pathology (BSK)	0.766	0.792	--													
4. Pharmacology (BSK)	0.721	0.802	0.779	--												
5. Physiology (BSK)	0.815	0.813	0.763	0.779	--											
6. CSK1	0.525	0.550	0.534	0.599	0.564	--										
7. CSK2	0.529	0.527	0.544	0.585	0.521	0.558	--									
8. CSK3	0.539	0.540	0.570	0.565	0.553	0.553	0.508	--								
9. CSK4	0.567	0.590	0.600	0.640	0.582	0.616	0.571	0.572	--							
10. CSK5	0.631	0.623	0.678	0.672	0.618	0.600	0.572	0.572	0.616	--						
11. Anatomy (VEA®)	0.493	0.383	0.404	0.338	0.451	0.323	0.293	0.330	0.329	0.350	--					
12. Physiology (VEA®)	0.429	0.425	0.424	0.404	0.479	0.321	0.271	0.332	0.322	0.355	0.572	--				
13. Pharmacology (VEA®)	0.337	0.362	0.330	0.351	0.375	0.318	0.243	0.277	0.297	0.357	0.450	0.594	--			
14. Microbiology (VEA®)	0.358	0.374	0.366	0.340	0.368	0.283	0.286	0.286	0.292	0.315	0.437	0.544	0.517	--		
15. Pathology (VEA®)	0.455	0.447	0.501	0.437	0.471	0.366	0.318	0.358	0.367	0.418	0.594	0.673	0.598	0.634	--	
16 NAVLE®	0.567	0.585	0.626	0.566	0.587	0.503	0.469	0.489	0.544	0.579	0.463	0.501	0.492	0.477	0.580	--
Skewness	− 0.258	− 0.368	− 0.415	− 0.239	− 0.210	− 0.243	− 0.343	− 0.350	− 0.319	− 0.234	− 0.020	− 0.218	− 0.259	− 0.020	− 0.108	− 0.073
Kurtosis	− 0.547	− 0.665	− 0.653	− 0.588	− 0.774	− 0.432	− 0.372	− 0.207	− 0.431	0.058	0.167	− 0.061	− 0.281	− 0.255	− 0.394	0.199

All correlations were significant (*p* <.01)

### Confirmatory factor analysis (CFA)

**Fig. 2:**
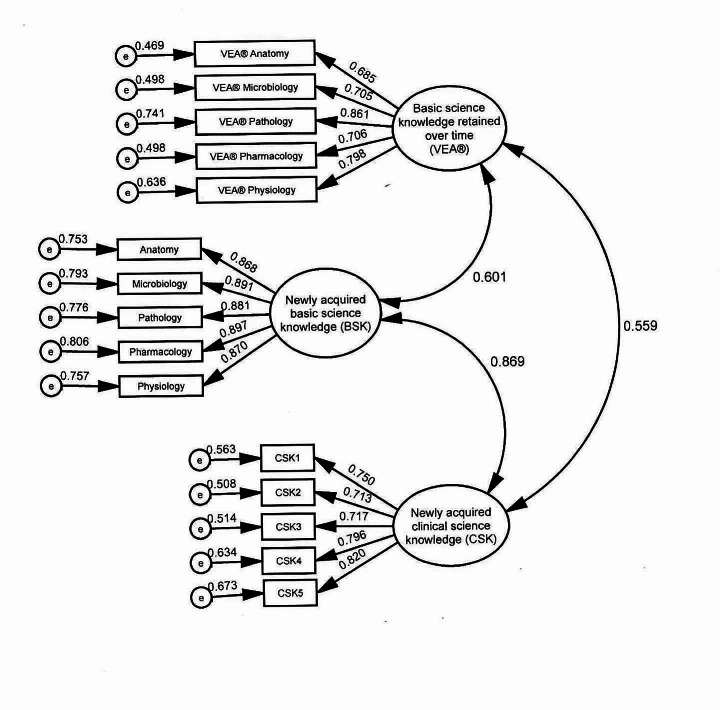
Confirmatory factor analysis. Newly acquired basic science knowledge (BSK) represented course grades in the subjects shown. Newly acquired clinical science knowledge (CSK) was measured with student achievement in clinical courses. e = error, VEA® = Veterinary Educational Assessment

As is common to SEM, CFA was conducted to verify the measurement quality of the latent constructs used in the model. Inspection of fit indices for the model presented in Fig. 2 showed that the model provided a good fit for the data, χ^2^(83) = 358.430, *p* <.001; RMSEA = 0.053, 90% CI [0.048, 0.059], *p* =.151; CFI = 0.978; TLI = 0.972; SRMR = 0.026. Table 2 presents the unstandardized and standardized coefficients, as well as the squared multiple correlation (SMC). As seen in Table 2, all indicators demonstrated significant loadings (e.g. standardized regression coefficients) on the expected factors. Additionally, inspection of the SMCs showed that BSK accounted for 75–81% of the variance in the indicators; CSK accounted for 51–67% of the variance in the indicators and VEA® accounted for 47–74% of the variance in the indicators. The composite reliability / McDonald omega coefficient (ω) for BSK was 0.943, 95% CI [0.935, 0.949], demonstrating excellent reliability. For CSK, ω = 0.876, 95% CI [0.863, 0.878], demonstrating good reliability. For VEA®, ω = 0.867, 95% CI [0.854, 0.878], also demonstrating good reliability.

**Table 2:** Unstandardized and standardized regression coefficients, confirmatory factor analysis

Indicator	B	SE	*p*	β	SMC
BSK					
Physiology	1.000			0.870	0.636
Pharmacology	1.019	0.024	< 0.001	0.897	0.498
Pathology	1.010	0.024	< 0.001	0.881	0.741
Microbiology	1.014	0.022	< 0.001	0.891	0.498
Anatomy	0.992	0.022	< 0.001	0.868	0.469
*CSK*					
CSK1	1.000			0.750	0.563
CSK2	0.964	0.040	< 0.001	0.713	0.508
CSK3	0.957	0.039	< 0.001	0.717	0.514
CSK4	1.060	0.039	< 0.001	0.796	0.634
CSK5	1.089	0.039	< 0.001	0.820	0.673
*VEA®*					
Physiology	0.926	0.030	< 0.001	0.798	0.757
Pharmacology	0.820	0.031	< 0.001	0.706	0.806
Pathology	1.000			0.861	0.776
Microbiology	0.819	0.031	< 0.001	0.705	0.793
Anatomy	0.796	0.031	< 0.001	0.685	0.753

B = unstandardized coefficient; SE = standard error; *p* = probability value, ≤ 0.05 considered significant; *β* = standardized coefficient; SMC = squared multiple correlation

### Structural equation models (SEM)

**Fig. 3:**
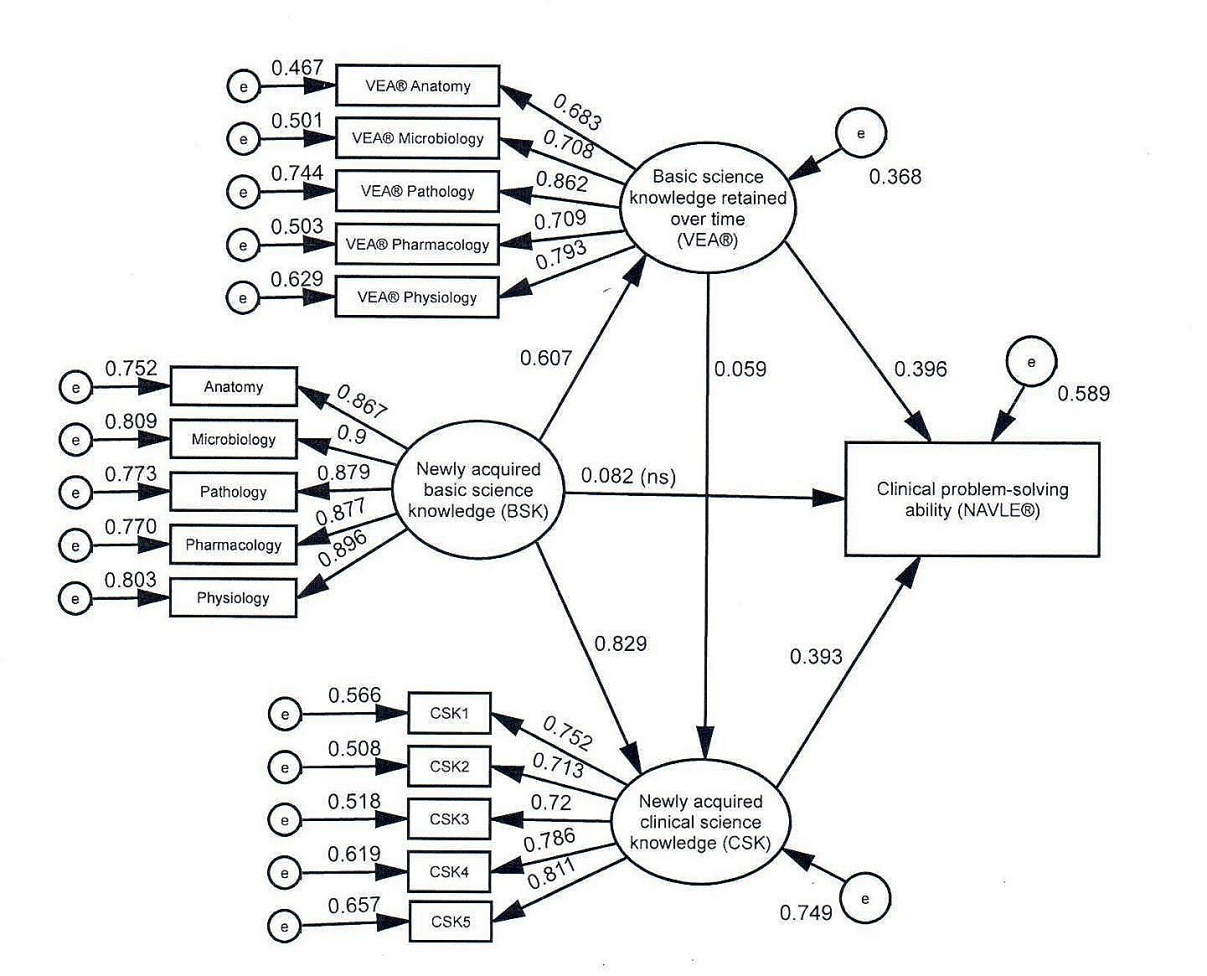
Structural equation model tested. Newly acquired basic science knowledge (BSK) represented course grades in the subjects shown. Newly acquired clinical science knowledge (CSK) was measured with student achievement in clinical courses. e = error, VEA® = Veterinary Educational Assessment, NAVLE® = North American Veterinary Licensing Examination

*Direct effects* Fig. 3 shows the model tested. In this model, BSK was hypothesized to predict CSK, VEA®, and NAVLE®. VEA® was hypothesized to predict CSK and NAVLE®. CSK was hypothesized to predict NAVLE®. Inspection of fit indexes for this model showed that the model provided a good fit for the data, χ^2^(99) = 509.651, *p* <.001; RMSEA = 0.060, 90% CI [0.055, 0.065], *p* =.001; CFI = 0.970; TLI = 0.963; SRMR = 0.026). Examination of the standardized residual covariance matrix showed that no covariance was greater than 2, indicating a good fit by conventional standards. Squared multiple correlations demonstrated that 37% of the variance in VEA®, 59% of the variance in NAVLE®, and 75% of variance in CSK were accounted for by the model.

Table 3 presents the unstandardized and standardized coefficients for the direct effects in the hypothesized model. As hypothesized, BSK predicted CSK, β = 0.829, and BSK predicted VEA®, β = 0.607. VEA® predicted CSK, β = 0.059, and NAVLE®, β = 0.396. CSK predicted NAVLE®, β = 0.393. BSK did not predict NAVLE®, β = 0.082, *p* =.120.

**Table 3:** Unstandardized and standardized regression coefficients, structural equation modeling direct effects

Path	B	SE	*p*	β
BSK → CSK	0.687	0.028	< 0.001	0.829
BSK → VEA®	0.586	0.029	< 0.001	0.607
BSK → NAVLE®	0.092	0.059	0.120	0.082
VEA® → CSK	0.050	0.023	0.031	0.059
VEA® → NAVLE®	0.459	0.031	< 0.001	0.396
CSK → NAVLE®	0.530	0.072	< 0.001	0.393

B = unstandardized coefficient; SE = standard error; *p* = probability value, ≤ 0.05 considered significant; *β* = standardized coefficient

*Specific indirect effects* Table 4 contains the specific indirect effects (SIE) of mediated variables. BSK’s effect on NAVLE® is mediated by VEA®, SIE = 0.269. BSK’s effect on NAVLE® is also mediated by CSK, SIE = 0.364. Finally, BSK’s effect on NAVLE® is mediated by both VEA® and CSK combined (see Fig. 3), SIE = 0.027.

*Individual school models* To explore the possibility that the tested model was a good fit for the multi-institution pooled data set, but not a good fit for one or more of the participating colleges, the same model was applied individually to each college. The significance of the coefficients for the direct and indirect relationships were identical in each of the individual college models with two exceptions. First, the direct relationship between VEA® and CSK was not significant in any of the four individual school models, and second, the direct relationship between CSK and NAVLE® was not significant for one of the individual school models. In both cases, because the underlying coefficients were similar across individual schools, we hypothesize that the relatively smaller institution samples provided inadequate statistical power to detect significant differences in the smaller single institution samples.

**Table 4:** Specific indirect effects (SIE)

Path	SIE, unstandardized	95% CI	*p*	SIE, standardized
BSK → VEA® → NAVLE®	0.269	0.227–0.318	0.001	0.415
BSK → CSK → NAVLE®	0.364	0.271–0.479	0.001	0.326
BSK → VEA® → CSK → NAVLE®	0.027	0.004–0.054	0.030	0.014

*p* ≤.05 considered significant

## Discussion

All of the hypotheses were supported by the findings, except the hypothesis of a direct positive relationship between newly acquired basic science knowledge and clinical problem-solving ability (NAVLE).

Based on prior research, we expected both measures of basic science knowledge to have a positive association with subsequent clinical science knowledge (Cianciolo et al., 2013; Danielson & Burzette, 2020; Danielson et al., 2011). The present study reinforces those findings and supports Schauber and colleagues’ (2013) hypothesis that their findings of a negative association were atypical.

In the present study, newly acquired basic science knowledge directly and significantly predicted both basic science knowledge retained over time and newly acquired clinical science knowledge, but did not directly predict clinical problem-solving ability. However, its association with clinical problem-solving ability was measurable when mediated by both CSK and VEA® independently, as well as by those two measures combined. This suggests three independent knowledge mechanisms by which BSK might be hypothesized to influence clinical problem solving. First, BSK’s direct association with CSK as well as its indirect association with NAVLE® (mediated by CSK) may represent the encapsulated basic science knowledge hypothesized to be integrated into clinical knowledge (de Bruin et al., 2005). Second, BSK’s direct association with VEA®, as well as VEA®’s association with CSK and NAVLE® likely represents the explicit knowledge of basic science concepts and principles that remain accessible to learners long after initial learning, and even after expertise has developed. The strength of these associations suggests that explicit basic science knowledge might be hypothesized to continue positively to affect clinical science knowledge, independent of encapsulated knowledge. The effect of BSK on NAVLE® that is mediated by both CSK and VEA® might be explained by some more general phenomenon that would contribute equally to both basic and clinical science knowledge, such as general intelligence or scientific reasoning. General scholastic ability, measured by instruments such as the ACT, SAT and GRE has been shown to be strongly related to general cognitive ability (Frey & Detterman, 2004; Koenig et al., 2008), and to scientific reasoning (Sternberg et al., 2019) when the latter was measured using a multiple choice format. Furthermore, within veterinary medicine, general scholastic ability, as measured by GRE verbal scores, have been shown to be significantly related to both VEA® and NAVLE® scores (Danielson & Burzette, 2020). Therefore, it is reasonable to hypothesize that general cognitive ability might contribute to achievement in both the basic and clinical sciences, and could explain the variance in NAVLE® scores identified in our study that is not explicitly linked to either basic or clinical science knowledge.

While these findings support the hypothesized relationships among basic and clinical science knowledge and clinical problem-solving ability, it would be naïve to suppose that any given measure of medical science ability reflects only basic science knowledge or clinical science knowledge. Most medical science learners have at least some exposure to both biomedical science and clinical knowledge very early in their educational process, and some veterinary learners have years of exposure to clinical cases prior to even enrolling in a veterinary program. Therefore, these findings likely suggest a gradual development of schema (encapsulated knowledge), and not the isolated development of biomedical vs. clinical knowledge over time, nor the point at which biomedical science ceases to contribute to achievement and clinical knowledge begins to contribute to achievement (see Rikers, Schmidt, et al., 2005). The fact that the predictive relationships were similar across multiple colleges with variability in learners, instructors, courses, assessments, and instructional/curricular approaches suggests that the relationships among variables, and any resulting theoretical implications, are resistant to such variability.

### Implications for instruction

A number of studies (Baghdady et al., 2009, 2013; Woods et al., 2005, 2006, 2006b, 2007) have identified a significant positive relationship between teaching strategies that explicitly teach relevant basic science concepts and subsequent clinical knowledge over a relatively short time frame (such as a week.) Given the results of the present study, such strategies may also have positive effects in the longer term. However, the present findings emerged from four institutions employing different curricular approaches, and from many instructors employing a broad variety of instructional and assessment approaches. Therefore, the broader implication is that explicitly teaching basic science knowledge might be hypothesized to positively and durably affect subsequent clinical knowledge independent of instructional strategy or curricular approach. Furthermore, for veterinary colleges specifically, student performance as measured by both course-level and standardized tests (such as the VEA®) are likely to prove useful for predicting subsequent academic achievement in both classroom and clinical settings, as well as licensing examination performance) and/or for identifying students likely in need of remediation in clinical knowledge.

### Limitations

This study was conducted at four veterinary colleges in North America. While those colleges’ curricular approaches varied, they were all inherently discipline based, and traditional in their approach; other common broad curricular approaches, such as Problem Based Learning (PBL), an organ-system based model or a clinical presentation-based model, were not represented. It is possible that these findings might not generalize to institutions that use substantially different curricular models, particularly models that seek to integrate basic and clinical science knowledge throughout the curriculum. Nonetheless, ample evidence suggests that curricular approach is a relatively weak factor for explaining student achievement, when compared with other factors, such as student or teacher effects (Hattie, 2015; Hecker & Violato, 2008), so implications regarding the generalizability of these findings due to curricular approach are likely to be modest. One strength of this study, the broad sampling of scores across several institutions and multiple courses, is also a potential weakness. While it allows us to conclude that basic science knowledge is important for clinical science knowledge, it does not provide detail regarding which instructional approaches may be more effective or less effective, or how much basic science knowledge is needed for subsequent clinical proficiency. Additionally, the present study was not able to partition general intelligence or aptitude prior to any of the basic knowledge instruction. Such factors have the potential to contribute to initial clinical or basic science knowledge or could influence the acquisition of knowledge or skills. Finally, clinical knowledge and problem solving, as defined in this study, do not include many important elements of a graduate veterinarian’s portfolio of abilities and attributes, including technical and other procedural skills, communication ability, professionalism, ethical conduct, time management and many context-specific abilities. Further research is recommended to address these limitations.

## Conclusion

The present study supports prior research documenting the positive predictive relationship between basic science knowledge and clinical problem solving. Specifically, basic science knowledge appears to contribute to clinical problem-solving ability both directly and mediated through subsequent clinical knowledge, where it is theorized to become encapsulated or schematized. While no specific instructional approaches are indicated based on the present study, this relationship between basic science and clinical science knowledge appears sufficiently robust to be evident across a variety of institutions, courses, instructors, and assessment processes.

Further research into the relationship between basic science knowledge and subsequent achievement is warranted. First, while this paper has documented the relationship between basic science knowledge, clinical science knowledge and clinical problem solving as defined narrowly, associations between these constructs and measures of workplace based clinical proficiency, including abilities such as procedural knowledge and skill, communication ability, time management, patient handling, and so forth, remain largely unexplored. Additional research to establish valid measures of workplace based clinical proficiency, and to associate those measures with predictive measures such as those discussed in this paper, will be critical to a growing understanding of how best to adapt educational practices to the needs of clinical practitioners.
